# Effects of Exocellobiohydrolase CBHA on Fermentation of Tobacco Leaves

**DOI:** 10.4014/jmb.2404.04028

**Published:** 2024-06-19

**Authors:** Xueqin Xu, Qianqian Wang, Longyan Yang, Zhiyan Chen, Yun Zhou, Hui Feng, Peng Zhang, Jie Wang

**Affiliations:** 1 China Tobacco Guangxi Industrial Co., Ltd., P.R. China; 2 Pest Integrated Management Key Laboratory of China Tobacco, Tobacco Research Institute of Chinese Academy of Agricultural Sciences, Qingdao, 266101, P. R. China

**Keywords:** CBHA, starch, cellulose, total nitrogen, microbial community

## Abstract

The quality of tobacco is directly affected by macromolecular content, fermentation is an effective method to improve biochemical properties. In this study, we utilized CBHA (cellobiohydrolase A) glycosylase, which was expressed by *Pichia pastoris*, as an additive for fermentation. The contents of main chemical components of tobacco leaves after fermentation were determined, and the changes of microbial community structure and abundance in tobacco leaves during fermentation were analyzed. The relationship between chemical composition and changes in microbial composition was investigated, and the function of bacteria and fungi in fermentation was predicted to identify possible metabolic pathways. After 48 h of CBHA fermentation, the contents of starch, cellulose and total nitrogen in tobacco leaf decreased by 17.60%, 28.91% and 16.05%, respectively. The microbial community structure changed significantly, with *Aspergillus* abundance decreasing significantly, while *Filobasidum*, *Cladosporium*, *Bullera*, *Komagataella*, etc., increased in CBHA treated group. Soluble sugar was most affected by microbial community in tobacco leaves, which was negatively correlated with starch, cellulose and total nitrogen. During the fermentation process, the relative abundance of metabolism-related functional genes increased, and the expressions of cellulase and endopeptidase also increased. The results showed that the changes of bacterial community and dominant microbial community on tobacco leaves affected the content of chemical components in tobacco leaves, and adding CBHA for fermentation had a positive effect on improving the quality of tobacco leaves.

## Introduction

Tobacco is considered as one of the important industrial and economic crops [1]. However, the quality of tobacco is directly affected by chemical indicators such as nitrogen, sugar, organic acids and aroma components [2]. The contents of nitrogen, sugar, and organic acids are related to sweetness and mellowness. Starch and cellulose, which belong to polysaccharides, are the basic components of tobacco leaves. Tobacco leaves are reported to contain approximately 50% carbohydrates (10-30% starch, 10-25% cellulose and 12% pectin) and 5-15% protein, which affects the quality of cigarettes [3]. High starch content of tobacco not only hinders burning and smoking, but also produce burnt taste and affect the user's senses [3-5]. The high content of protein in tobacco leaves will not only cause the odor of burnt feathers, but also reduce the burning property of cigarettes, increase the content of harmful components, and seriously affect the quality and safety of cigarettes [3-5]. High cellulose (cell wall material) content makes tobacco leaves coarser and brittle, and smoke irritants strong, reducing tobacco flavor and smoke quality [3-5]. Therefore, proper degradation of starch, cellulose and protein is the key to improve the quality of tobacco leaves.

Based on the above, freshly harvested tobacco leaves have a sharp odor and undesirable aroma as well as pungent, irritating smoke, and are not suitable for cigarette products [6]. Tobacco leaves contain many microorganisms, and the fermentation process of tobacco is complex, involving the enzymatic action of microorganisms and chemical interaction in tobacco leaves. The microbial community and its activities are of great significance to improve tobacco quality. Liu *et al*. found that during the fermentation process, the community structure of bacteria and fungi changed significantly, and the chemical composition was affected by microbial activity [7]. In recent years, high-throughput sequencing techniques based on 16S rRNA gene sequences have been used to analyze the diversity and dynamics of microbial communities in different types of tobacco [6, 8, 9]. Theoretically, fermentation can affect the growth and metabolism of microbial community, change the biochemical reaction pathway, and thus improve the quality of flue-cured tobacco. However, little is known about the microorganisms appropriate to assist tobacco fermentation and their role in tobacco fermentation [5, 7, 10, 11].

Tobacco fermentation includes artificial fermentation and natural aging. Compared with natural aging, artificial fermentation can significantly shorten the fermentation period by controlling moisture and temperature [7]. Based on this trend, exogenous additives are proposed to promote the fermentation process. Exogenous additives currently used in tobacco fermentation includes plant extracts, microorganisms, enzyme preparations, and mixtures of the above materials [12]. Biological enzyme preparations are environmentally friendly, non-toxic biocatalesters that can promote most reactions in biological systems, including proteins and a small part of RNA. They have special catalytic functions such as specificity, low reaction conditions, high efficiency, and can reduce the activation energy of biochemical reactions [13]. For example, treating low-grade tobacco leaves with amylase and protease can reduce the irritating burnt odor during combustion and improve the availability and economic benefits of tobacco leaves.

For cellulose, β-1,4-glucanases or cellobiohydrolases act on reducing and non-reducing chain ends to produce cellobiose from cellulose fragments. Then cellobiose and other low molar mass oligosaccharides are converted to glucose [14-16]. Although most commercial plant cell-wall-degrading enzyme preparations used today are derived from fungi, the cellulosomal enzyme system from Clostridium thermocellum is an equally effective catalyst, yet of considerably different structure. Bacterial extracellular glycoside hydrolases can hydrolyze β1,4 glucan to reducing sugars, such as β-1,4-cellobiohydrolase A (CBHA), which active against heterogeneous plant cell wall materials [14-16]. The *CBHA* gene of cellulolytic bacterium *Cellulomonas fimi* encodes a protein of 872 amino acids and has a predicted molecular mass of 85,349 Da. [15]. As a fibrinolytic enzyme, CBHA is a large seven-module enzyme with a catalytic module belonging to family 9. In contrast to other representatives of this family, which has only endo-cellulase activity and, in a few cases, endo-/exo-cellulase activity, CBHA is entirely an exo-cellulase [17].

In this paper, we used the glycosyl hydrolase CBHA as an exogenous additive to ferment tobacco. The CBHA was expressed with *P. pastoris* X-33 expression system. The *P. pastoris* expression system has many advantages, such as the target product can be secreted outside the cell, the endogenous secreted proteins are less than the bacterial expression system, and it is easy to purify. At the same time, compared with bacteria, it also has the advantages of fast growth rate, modification after scouring, and easy genetic manipulation. In addition, the linear DNA can be efficiently inserted into the chromosomes of yeast cells by cross-recombination, thus producing stable cell lines [18-20]. The supernatant of yeast medium after induction was used to ferment low grade tobacco leaves planted in Guangxi, China. The effects of these exogenous additives on the bacterial community structure and function were studied by biochemical means and high throughput sequencing analysis.

## Materials and Methods

### The Expression of CBHA in *P. pastoris*

Fusion vector containing CBHA gene sequence and the strong methanol-inducible AOX1 promoter were transformed into *P. pastoris* X-33 cells to generate recombinant glycosyl hydrolase CBHA [21]. *P. pastoris* cells were cultured in 500 ml BMMY liquid medium at 28°C, 180 rpm for 4 days. 0.5% methanol was added to the medium every 24 h to induce the expression of CBHA. 100 ml of fermentation broth induced for 0, 1, 2, 3 and 4 d were collected, centrifuged at 8,000 ×*g* at 4°C for 20 min, and discarded the precipitation. The crude enzymes in the supernatant were precipitated with 80% ammonium sulfate. Centrifuge at 4°C 8,000 ×*g* for 20 min and discard the supernatant. The precipitated protein is resuspended in 1mL buffer with 20 mM HEPES, 50 mM MgCl_2_, pH 7.5. The concentrated CBHA was obtained by overnight dialysis with a dialysis bag. The concentrated protein was detected by sodium dodecyl sulfate -polyacrylamide gel electrophoresis (SDS-PAGE) and Western blot analysis.

### SDS-PAGE and Western Blot

SDS-PAGE was carried out using 8% polyacrylamide gels, described as [22]. In order to conduct electrophoresis, CBHA protein was initially denatured with 5% [v/v] β-mercaptoethanol at 95°C for 10 min. The mixture was then run at 120 V until the dye front reached the bottom. Western blot was performed as detailed previously [23].

### Determination of Fermentation Conditions

The *P. pastoris* suspension induced by methanol was collected and the precipitate was removed by centrifugation. The supernatant was mixed with a ratio of 20 ml per 100 g tobacco leaves. The treatments were applied to the tobacco leaves by foliar spray while they were soaking wet. The sprayed tobacco leaves were then placed in an incubator with a humidity of 60% and a temperature of 28°C. The leaves were fermented for different durations of 0, 3, 6, 12, 24, 36, 48, 72, 96, and 120 h. The tobacco leaves treated without supernatant were used as negative controls.

### Determination of Chemical Components

Six conventional chemical components in tobacco, including starch, cellulose, pectin, soluble sugar, protein and total nitrogen, were usually of general concern in the tobacco industry. Thus, contents of the above-mentioned chemical components were analyzed in this study firstly. The soluble sugar, protein and starch were determined using Plant Soluble Sugar Content Detection Kit, Bradford protein assay kits and Starch Content Detection Kit (Beijing Solarbio Science & Technology Co., Ltd., China), respectively. The total nitrogen content was determined by Shimadzu Total Nitrogen analyzer (Model: TOC-VCPH) [24]. Cellulose content in the tobacco leaves was detected by the content detection Kit (Solarbio BC4280). The content of pectin was detected by the total pectin content kit of Suzhou Keming Biotechnology Co., Ltd. (ZGJ-1-G).

### DNA Extraction and PCR-DGGE (Denaturing Gradient Gel Electrophoresis) Analysis

Microbial DNA was then isolated using the Qiagen DNeasy Microbial DNA Extraction Kit (QIAGEN Co.,Ltd., Germany) according to the manufacturer's instructions. The integrity and concentration of the extracted DNA were determined by 1% agarose gel electrophoresis and a Nanodrop 1000 spectrophotometer (Thermo Fisher Scientific, USA) [25]. For PCR-DGGE, the PCR program was set at 94°C for 2 min, followed by 35 cycles of 94°C for 10 s, 56°C for 10 s, and 72°C for 30 s. The primers used were F341GC (5'- CGCCCGCCGCGCCCCGCGCCC GGCCCGCCGCCCCCGCCCCCTACGGGAGGCAGCAGCCTACGGGAGGCAGCAG -3') and R518 (5'- ATT ACCGCGGCTGCTGG -3'). The diversity of bands was detected with 1% agarose gel.

### High throughput Sequencing and Bioinformatics Analysis

The V3-V4 region of the bacterial 16S rRNA gene was amplified with primers 338F (5'-ACTCCTACGGGA GGCAGCAGCAGG- 3') and 806R (5'-GACTACHVGGGTWTCTAAT- 3') [12]. The fungi internal transcribed spacer (ITS) gene was amplified using primers ITS1F (5'-CTTGGTCATTTAGAGGAAGTAA-3') and ITS2R (5'-GCTGCGTTTCTTTCATCGATGC-3') [12]. PCR products were sequenced using the Illumina MiSeq platform Majorbio Bio-Pharm Technology Co., Ltd. China). For sequence processing, FASTP (0.19.6) and FLASH (v.1.2.11) were used for quality control. The α-diversity of microbial community was canculated and analyzed by MOTHUR (v.1.30.0) software [26]. Community barplot or heatmap and β-diversity analysis was performed with R (v.3.3.1) package.

### Statistical Analysis

All experiments were performed in three replicates. The means and standard deviation (SD) values are shown in the figures. Statistical differences among multiple groups were employed by One-way ANOVA and Student’s *t*-test was applied to determine that between two groups: *p < 0.05, **p < 0.01, *t*-test.

## Results and Discussion

### Changes of Major Chemical Component after Fermentation

**Fig. 1:**
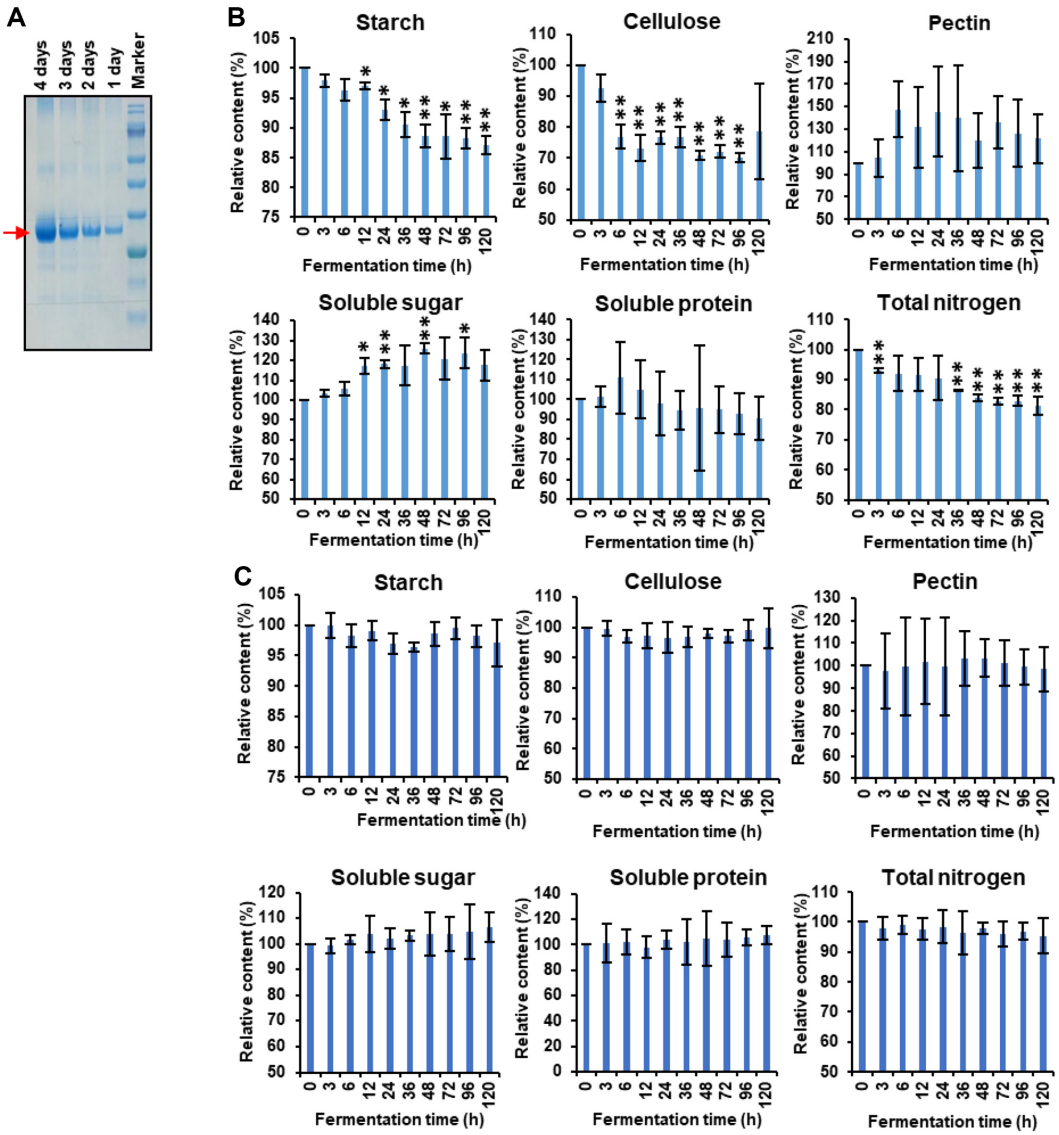
Expression of CBHA and its degradation function of macromolecular substances in tobacco leaves. (**A**) SDS-PAGE detection of *P. pastoris* expression protein CBHA of 1, 2, 3 and 4 days after induction. (**B** and **C**) Changes of starch, cellulose, pectin, soluble sugar, soluble protein and total nitrogen with (**B**) or without (**C**) CBHA treatment. Data are expressed as means ± SD of three independent biological replicates. Significant differences from Col-0 were determined by Student’s *t*-test: *p < 0.05, **p < 0.01, *t*-test.

The high content of macromolecules in flue-cured tobacco is one of the main causes of producing impurities and irritation by reducing the mellow, fluency, lingering, sweetness, cleanliness and aftertaste [4, 5]. Besides of variety optimization, fermentation is the most effective approach to improve tobacco quality. In this study, CBHA, a Xyloglucan-specific exocellobiohydrolase, was used as a novel additive for tobacco fermentation [27, 28]. In order to obtain high expression of cellulose exonuclease, we used *P. pastoris* expression protein CBHA. The enzyme activity was detected by Cellulase Activity Assay Kit (Boxbio, China) before the tobacco fermentation. The results showed that the cellulase activity of CBHA sprayed on tobacco leaves was about 74.66 U/ml (Fig. S1). SDS-PAGE and Western blot were performed to detect the expression level of extracellular protein, and the bands of the corresponding size of the protein were indeed detected in the supernatant, indicating that the *P. Pastoris* expression system can effectively express CBHA protein and secrete it into the extracellular (Fig. 1A, Fig. S2). In order to verify the effect of CBHA on tobacco quality, the optimal solid fermentation time on the content of major macromolecular substances were studied first. The samples fermented for 0, 3, 6, 12, 24, 36, 48, 72, 96, and 120 h were analyzed for starch, cellulose, pectin, soluble sugar, protein and total nitrogen. The chemical composition of the tobacco leaves was changed by CBHA and microorganisms during the fermentation process (Fig. 1B). As shown in Fig. 1B, at the early stage of CBHA treatment (0-12 h), cellulose content decreased significantly. At 48 h, the soluble sugar content increased to the maximum, the content increased by 23.76%, and the cellulose degradation rate was about 28.91%. This may be the role of CBHA in the degradation of cellulose and hemicellulose in tobacco cell wall as glycosylase. The cellulose content also decreased until 96 h of fermentation then began to increase, and the total sugar content began to decrease slowly after 48 h (Fig. 1B). These results showed that longer fermentation time was not the better, and it was necessary to explore different optimum fermentation time in different medium.

Tian *et al*. found that the addition of cellulase could reduce the content of starch when investigated the effects of different additives on the fermentation quality, nutrient composition, bacterial communities, and metabolic profiles of the silage of hybrid *Pennisetu* [29]. It was reported that cellulase contributes more to starch liberation than pectinase during cassava pulp hydrolysis [30]. In this paper, the content of starch also decreased significantly after 48 h, and decreased by 17.60% compared with the control group (0 h). These results indicated that CBHA as an additive in tobacco fermentation could improve the biodegradation efficiency of starch and cellulose in tobacco and increased the sweetness of tobacco. We all know that cellulase is involved in the degradation of cell wall by hydrolyzing cellulose, and it is speculated that cellulase destroys the cell wall structure of grains and promotes the release of starch [31, 32]. Cellulose degradation supplies D -glucose, cellobiose and cellulose oligosaccharides to the microbial community, while starch is hydrolyzed to glucose by amylase. In order to further detect the types and quantities of soluble sugars, the contents of maltose, fructose and glucose in fermented tobacco were respectively detected. The study found that compared with the control, the content of glucose increased significantly after CBHA fermentation, while the fructose and maltose hardly changed (Fig. S3). Glucose is a widely consumed organic carbon source for many microorganisms [33]. The glucose showed the desired effect of increasing microbial activity.

As can be seen from Fig. 1B, the overall change of pectin and protein content in tobacco leaves was not significant. The content of total nitrogen decreased gradually with the extension of fermentation time. After fermentation for 120 h, it decreased by 18.67%. Total nitrogen is one of the most influential chemical components on sensory quality of tobacco leaves. Nitrogenous compounds in tobacco leaves include alkaloids, amino acids and proteins, nitrogenous heterocyclic compounds, amines and nitrosamines, nitrogenous pigments, nitrates, and Maillard reactants [34]. For the tobacco leaves fermented by CBHA, the total nitrogen content decreased significantly, while the protein content did not show regular fluctuations (Fig. 1B). It is speculated that the composition of alkaloids and other nitrogen-containing compounds decreased significantly. These compounds usually make tobacco taste bitter and rough, and the decrease of total nitrogen content contributes to the improvement of tobacco flavor quality and safety [35, 36]. The decrease of total nitrogen could reduce the irritation of tobacco. These results indicated that CBHA can be used as a fermentation additive of tobacco leaves.

### Differences in Microbial Diversity of Tobacco Leaves before and after CBHA Fermentation

**Fig. 2:**
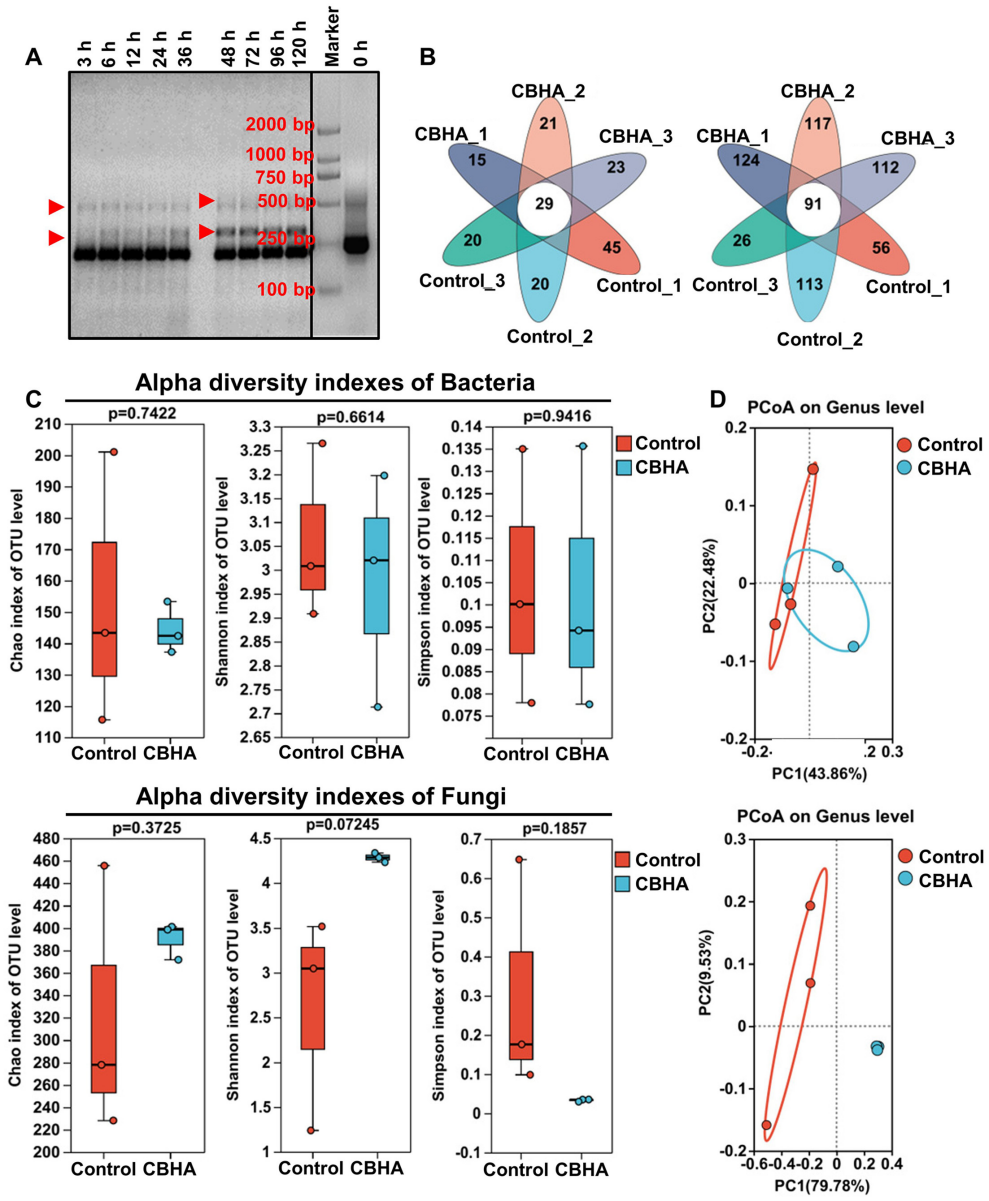
Effects of CBHA fermentation on microbial diversity of tobacco leaves. (**A**) DGGE fingerprints of microbial communities from tobacco leaves. The total microbial DNA was extracted and amplified by PCR with F341GC and R518 primers. The number of bands was counted and photographed. (**B**) Venn diagram of OTU distribution of bacterial (left) and fungal (right) communities. (**C**) Changes in αdiversity of microorganisms from tobacco leaves. (**D**) PCoA of bacteria (up) and fungi (down).

The fermentation technology and microbial community endow tobacco leaves with more flavors [37]. In order to study the effects of CBHA fermentation on microbial communities in tobacco, DGGE analysis was performed. The results showed that the microbial community was relatively low in complexity before fermentation, and a few species were dominant (Fig. 2A). The number and abundance of amplified bands increased with the extension of fermentation time, and the diversity remained constant between 48 and 120 h. At this time, the microbial diversity reached the highest, which was consistent with the results of macromolecular substance metabolism in the fermentation process (Figs. 1B and 2A), indicating that CBHA, as a new additive for tobacco fermentation, not only played the role of cell wall degrading, but also was beneficial to the growth of microorganisms in tobacco itself, which helped to accelerate tobacco fermentation and shorten the fermentation time. Considering that excessive fermentation is detrimental to the growth and metabolism of microorganisms in tobacco [12], 16S rRNA high-throughput sequencing technology was used to analyze the bacterial diversity after 48 h fermentation. The results showed that 49-74 bacterial OTUs (Operational Taxonomic Units) and 117-204 fungi OTUs were found in the control samples, and 44-52 bacterial OTUs and 203-215 fungi OTU were found in the CBHA fermentation samples (Fig. 2B). Apparently, there were 353 OTUs of unique fungi after fermentation, accounting for 55.24% of the total OTUs (Fig. 2B).

α-diversity analysis also showed that the Chao1, Simpson and Shannon indexes of bacteria were almost unchanged, while those of fungi increased (Fig. 2C). Chao1 index represents richness, while both Shannon index and Simpson index represent diversity [38]. Simpson index is negatively correlated with Shannon. As can be seen from the indexes, the abundance and diversity of bacteria were similar, while the fungi were slightly higher than those of the control group (Fig. 2C). PCoA (unconstrained Principal Coordinate Analysis) showed that the microorganisms with CBHA and those without CBHA were distributed in different quadrants and separated by the PC1 axis (Fig. 2D). The interpretation rate of PC1 axis isolation was 43.86% for bacteria and 79.78% for fungi (Fig. 2D). These results indicated that there were significant differences in the fungi and bacterial communities between the control and CBHA group, especially in the fungi communities.

### Analysis of Microbial Community Structure during Fermentation Process

**Fig. 3:**
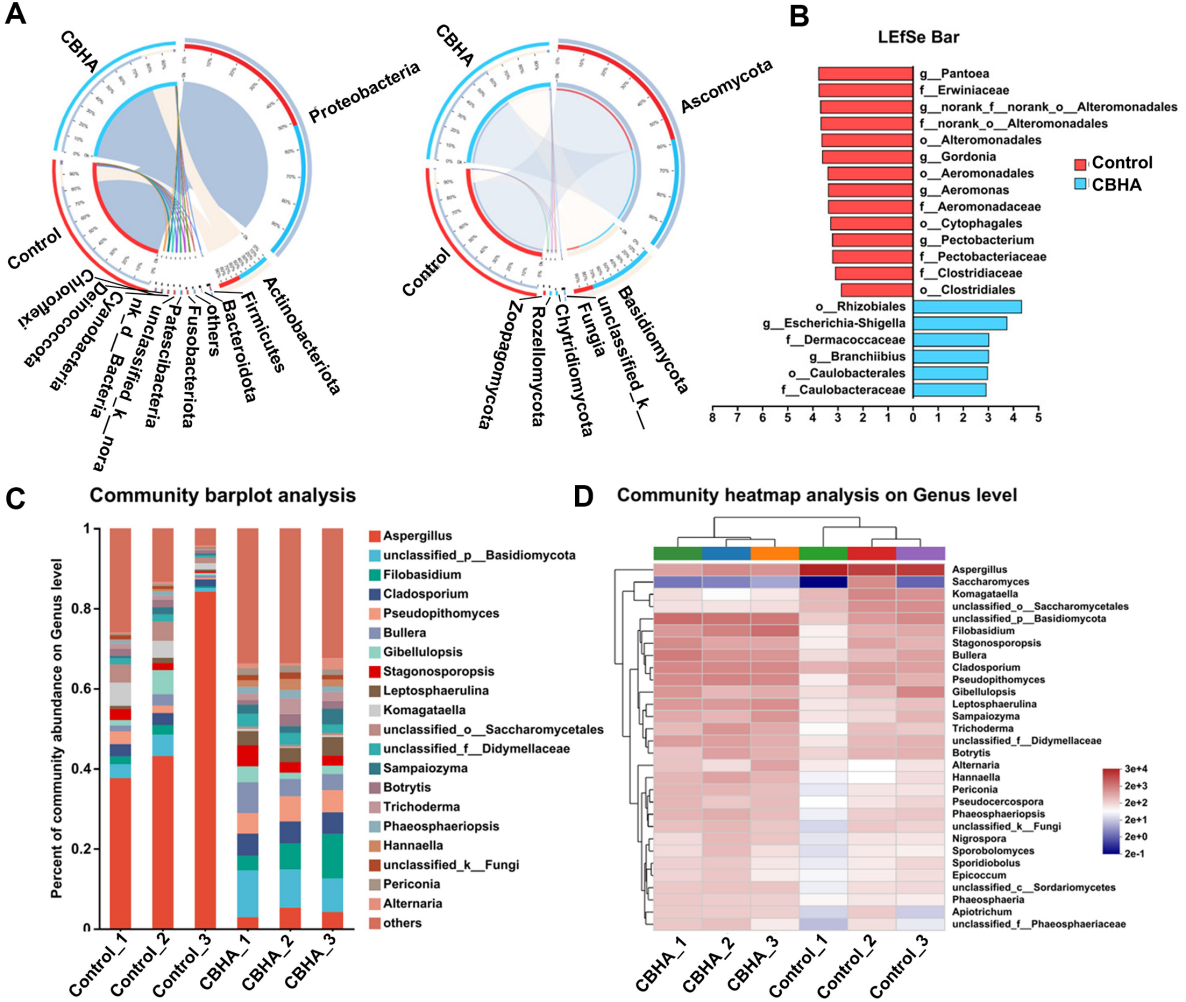
Analysis of microbial community composition during fermentation process. (**A**) Circos analysis of bacteria (left) and fungi (right) on phylum level from tobacco leaves after CBHA fermentation. (**B**) LEfSe results of bacteria biomarkers on genus level. (**C**) Community barplot analysis of fungi on genus level. (**D**) Heatmap of the fungi community on genus level.

Phylogenetic analysis showed that the identified sequence belonged to 11 phylum. From the average abundance of the first two dominant phylum, *Proteobacteria* accounted for 52% and 48% before and after fermentation, *Actinobacteriota* are 40% and 60%, respectively (Fig. 3A). The fungi identified mainly belonged to six phylum, with *Ascomycota* accounting for 59% and 41% and the *Basidiomycota* accounting for 22% and 77% in the control and treatment group, respectively (Fig. 3A). There was no doubt that fermentation changed the composition of the microbial community. *Rhizobiales* and *Escherichia-Shigella* were significantly increased by CBHA treatment at the genus level for bacteria community. However, *Pantoea*, *Erwiniaceae*, *Alteromonadales*, *Gordonia*, *Aeromonadales*, etc. were significantly reduced (Fig. 3B). The barplot of the community showed that CBHA treatment significantly changed the composition of the fungi community and increased the abundance at the genus level (Fig. 3C). Heat maps showed that for the first 30 fungi genus, *Aspergillus*, *Komagataella* and unclassified_o__*Saccharomycetales* decreased obviously, while the abundance of *Filobasidum*, *Cladosporium* and *Bullera* increased from 18%, 32%and 24% to 82%, 68% and 76%, respectively, which was consistent with the results in Fig. 3C (Fig. 3D). *Cladosporium* was used in the production of cellulase and protease [39]. *Bullera* always considered to be a producer of β-galactosidase, toxins, and coenzyme Q10 [40]. *Filobasidum* can produce extracellular amylase for starch degradation in tobacco leaves [41]. *Filobasidum* can also be used in the biosynthesis of perillolactone and its analogues, which are all premium tobacco flavoring agents [41]. The results showed that adding CBHA significantly improved the relative abundance and diversity of the fungi community.

### Relationship between Microbial Community and Core Metabolites

**Fig. 4:**
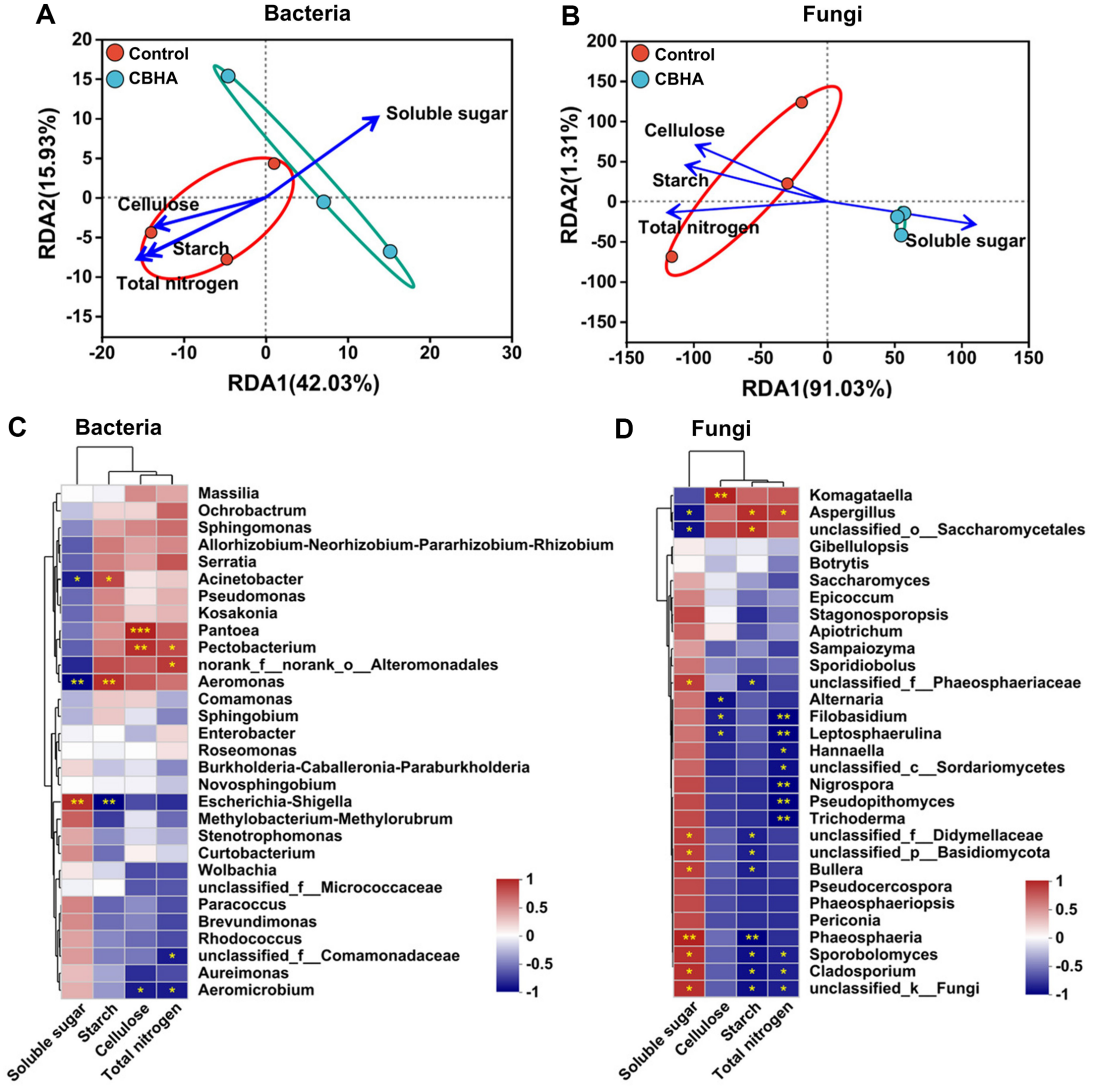
Correlation analysis of macromolecular content with microbial composition in tobacco leaves. (**A** and **B**) RDA of starch, cellulose, soluble sugar and total nitrogen with bacteria and fungi. (**C** and **D**) Heatmap analysis of correlation of starch, cellulose, soluble sugar and total nitrogen with bacteria (**C**) and fungi (**D**) in tobacco leaves. * Represents a significant difference at *p* < 0.05; ** represents a significant difference at *p* < 0.01; *** represents a significant difference at *p* < 0.001.

In order to detect the relationship between macromolecule content and microbial community, RDA (Redundancy Analysis) was performed. The RDA1 axis explained 42.03% and 91.03% of bacterial and fungi community data variation, respectively (Fig. 4A and 4B). Among all macromolecular substances, soluble sugar fermentation was most affected by bacterial and fungi communities in tobacco leaves (Fig. 4A and 4B). At the genus level, CBHA treatment was distributed around soluble sugar and far away from the source, indicating that CBHA treatment had the most significant effect on the generation of soluble sugar in tobacco leaf (Fig. 4A and 4B). Soluble sugars were negatively correlated with starch, cellulose and total nitrogen (Fig. 4A and 4B). These results indicated that CBHA treatment affected the microbial community during tobacco leaf fermentation, and the changes of microbial community further affected the transformation of macromolecular substances.

During co-culture, CBHA degrades cellulose in the cell wall of tobacco leaves to produce reducing sugars, and the surface of tobacco leaves and some endophytic microorganisms begin to reproduce using reducing sugars as energy. Interestingly, a noteworthy increase of reducing sugar contents was observed at an early stage during fermentation process, which highlighted the intense hydrolyzing activity of microbial community, and the rate of hydrolysis of substrates to reducing sugar is faster than the utilization of reducing sugar. Circos analysis from tobacco leaves after CBHA fermentation showed that *Basidiomycota* (decomposer of lignin) account for 22% and 77% in the control and treatment group (Fig. 3A), suggesting that microbial reproduction also contributes to sugar production [42]. At the class level, soluble sugar showed positive correlation with the abundance of *Agaricostilbomycetes*, *Cystobasidiomycetes*, *Dothideomycetes* and *Basidiomycetes*, and significantly positively related with *Eurotiomycetes*. For order, family and genus, we counted the top 30. At the order level, soluble sugar was positively correlated with 11 bacteria and fungi and negatively correlated with 2 bacteria and fungi. At the family level, soluble sugar was positively correlated with 11 bacteria and fungi and negatively correlated with 4 bacteria and fungi. At the genus level, soluble sugar was positively correlated with 9 bacteria and fungi and negatively correlated with 4 bacteria and fungi. In bacteria, soluble sugar was significantly negatively correlated with the relative abundance of *Acinetobacter* and *Aeromonas*, and significantly positively correlated with the abundance of *Escherichia-Shigella* (Fig. 4C). Increased *Acinetobacter* abundance may increase proteolytic activity in tobacco leaves (Fig. 4C and 4D)[43]. Soluble sugar showed negative correlation with *Aspergillus fungi* and unclassified_*o__Saccharomycetales*, and significantly positively related with *unclassified_f__Phaeosphaeriaceae*, unclassified_*f__Didymellaceae*, unclassified_*p*__*Basidiomycota*, *Bullera*, *Phaeosphaeria*, *SpoRobolomyces*, *Cladosporium* and unclassified_*k__Fungi* abundance (Fig. 4C and 4D). The microbial abundance affecting starch, cellulose and total nitrogen showed an opposite pattern to that affecting soluble sugar. Starch was positively correlated with 4 bacteria and fungi and negatively correlated with 9 bacteria and fungi (Fig. 4C and 4D). Cellulose was positively correlated with 3 bacteria and fungi and negatively correlated with 4 bacteria and fungi (Fig. 4C and 4D). Total nitrogen was positively correlated with 3 bacteria and fungi and negatively correlated with 12 bacteria and fungi (Fig. 4C and 4D).

### Prediction Analysis of Functional Genes of Microbial Metabolic Pathways

**Fig. 5:**
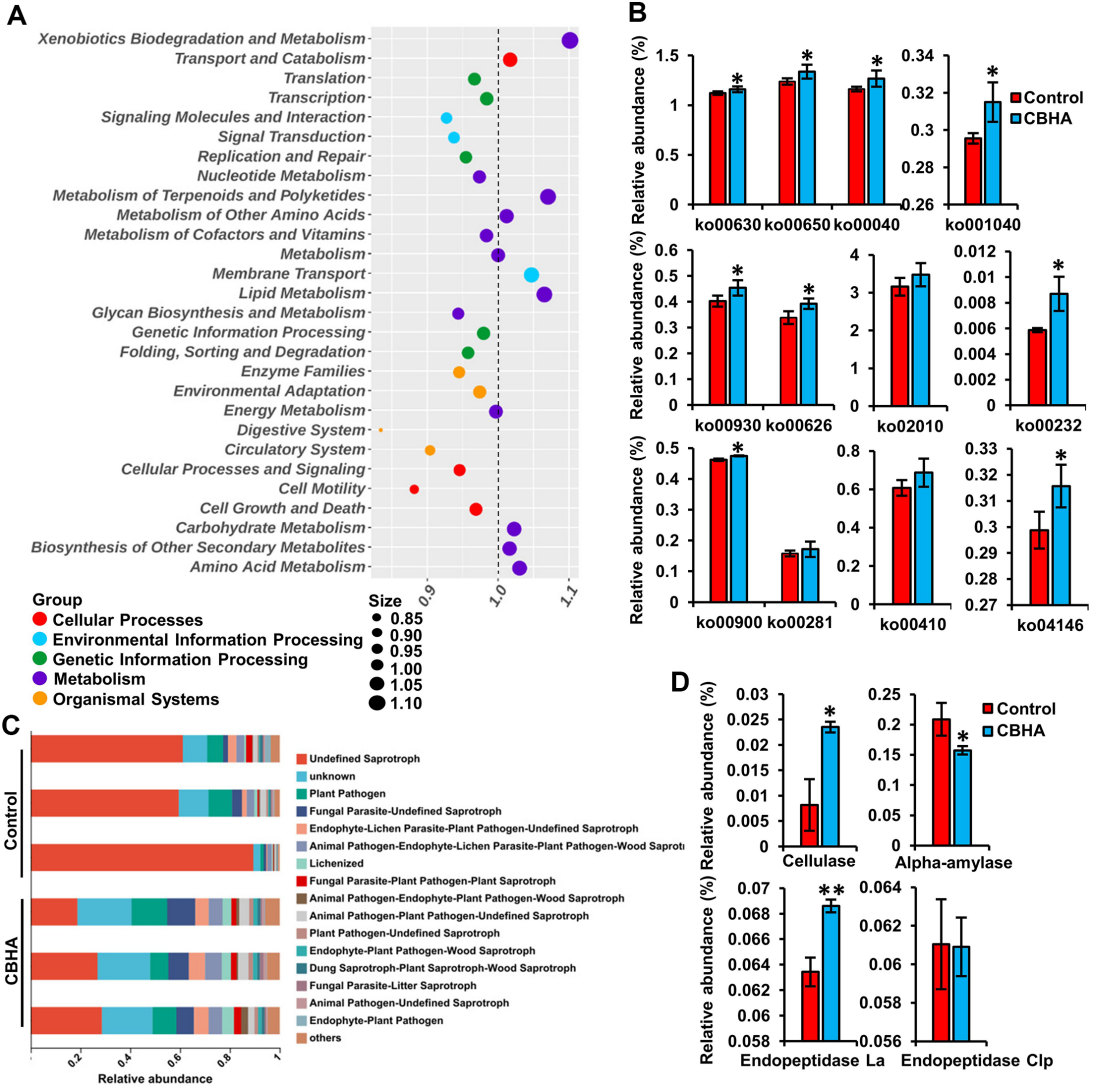
Prediction analysis of functional genes of microbial metabolic pathways. (**A**) The bubble map of the enrichment of the metabolite KEGG pathway in level 2. (**B**) Prediction of functions at level 3 of Xenobiotics Biodegradation and Metabolism (ko00930 and ko00626), Metabolism of Terpenoids and Polyketides (ko00900 and ko00281), Metabolism of Other Amino Acids (ko00410), Lipid Metabolism (ko00140), Carbohydrate Metabolism (ko00630, ko00650 and ko00040), Biosynthesis of Other Secondary Metabolites and Amino Acid Metabolism (ko00232). ko00630, glyoxylate and dicarboxylate metabolism; ko00650, butanoate metabolism; ko00040, pentose and glucuronate interconversions; ko00140, steroid hormone biosynthesis; ko00930, caprolactam degradation; ko00626, naphthalene degradation; ko02010, ABC transporters; ko00232, caffeine metabolism; ko00900, terpenoid backbone biosynthesis; ko00281, geraniol degradation; ko00410, β-alanine metabolism; ko04146, peroxisome. (**C**) Prediction analysis of functional genes of fungi metabolic pathways. (**D**). Relative abundance of enzyme. * Represents a significant difference at *p* < 0.05; ** represents a significant difference at *p* < 0.01.

The results of PICRUSt's bacterial function analysis showed that in level1, the main pathway were metabolism (76.35% for control vs. 77.00% for CBHA), genetic information processing (5.30% for control vs. 5.13% for CBHA), environmental information processing (6.18% for control vs. 6.14% for CBHA), cellular processes (5.21%for control vs. 5.04% for CBHA) and organic systems (2.03% for control vs. 2.05% for CBHA). Obviously, CBHA treatment mainly improved the metabolic capacity of bacteria, mainly manifested in the improvement of Xenobiotics Biodegradation and Metabolism, Metabolism of Terpenoids and Polyketides, Metabolism of Other Amino Acids, Lipid Metabolism, Carbohydrate Metabolism, Biosynthesis of Other Secondary Metabolites and Amino Acid Metabolism at KEGG level 2 pathway (Fig. 5A). Transport and catabolism and Xenobiotics Biodegradation and Metabolism may be related to lignin degradation and detoxification activity [44]. Abundant Xenobiotics Biodegradation and Metabolism function be conducive to phyllosphere health [45]. In addition, the other metabolisms related to predominant species, such as Metabolism of Terpenoids and Polyketides, also contribute to the flavor [46]. Terpenoids and polyketides are secondary metabolites with bioactive substances [47], increased abundance of Metabolism of Terpenoids and Polyketides, Lipid metabolism, Energy Metabolism, Amino Acid Metabolism indicated stronger cell viability of microorganisms. In addition, the Transport and Catabolism in Cellular Processes and the Membrane Transport in Environmental Information Processing increased significantly, indicating increased membrane transport and transport and catabolism after tobacco fermentation (Fig. 5A). The analysis at level 3 also showed that CBHA treatment significantly increased microbial metabolism, especially increased Styrene degradation, Ethylbenzene degradation, Linoleic acid metabolism, Glyoxylate and dicarboxylate metabolism, Pyruvate metabolism, Fructose and mannose metabolism, Limonene and pinene degradation, Geraniol degradation, D-Arginine and D-ornithine metabolism, ABC transporters, Endocytosis, Caffeine metabolism (Fig. 5B). The metabolism of ethylbenzene and styrene also contributes to the improvement of tobacco quality by reducing smoke during combustion [48]. It may be that the degrading of cell wall by CBHA could provide available nutrients for tobacco leaf microorganisms and enhanced cell metabolism. RDA analysis showed that starch, total nitrogen and other macromolecular substances were affected by bacterial and fungi communities in tobacco leaves (Fig. 4A and 4B).

The functions of fungi were studied using FUNGuild (Fungi Functional Guild), the function of fungi was predicted. Compared with the control group, the abundance of Undefined Saprotroph was reduced from 69.99%to 24.78% after CBHA treatment, indicating that CBHA could directly or indirectly inhibited the growth of saprophytic fungi during tobacco fermentation (Fig. 5C). Enzyme analysis by PICRUSt showed that the contents of cellulase and endopeptidase increased significantly, although the abundance of α-amylase decreased (Fig. 5D). ELISA analysis showed that the abundance of cellulase in tobacco leaves after CBHA fermentation was 12.3 times that of control, which was consistent with the predicted trend (Fig. S3). As we all know, starch degradation is either hydrolytic (via amylases) or phosphorolytic (via starch phosphorylases) [49]. The hydrolytic pathway of starch degradation involves α-amylase (AMY) and β-amylase (BAM). AMY is an endoamylolytic enzyme that specifically hydrolyses α-1,4-glucan bonds to yield various linear and branched malto-oligosaccharides. BAM belongs to the glycosyl hydrolase 14 family and hydrolyses α-1,4-linked glucan chains from the non-reducing end and catalyses the release of β-maltose [50]. It is believed that the major pathway of starch degradation occurs via BAMs in Arabidopsis and other organisms [49]. It has also been reported that pectin and cellulose intertwine starch to restrict α-amylase accessible [30]. Therefore, we speculated that the β-amylase hydrolysis pathway or phosphorylhydrolase pathway may play a major role in this process. In conclusion, the fermentation of CBHA not only changed the structure of the bacterial community, but also changed the function of the microbial community. This change may be conducive to the enhancement of tobacco flavor.

In conclusion, CBHA fermentation could significantly improve the quality of tobacco leaves. As cellobiohydrolase, CBHA can not only significantly reduce the cellulose content in tobacco leaves, but also improve the microbial metabolism by regulating the microbial community structure, which indirectly play important role in degradation of macromolecular such as starch and nitrogen-containing compounds in tobacco leaves. In addition to tobacco, straw also contains large amounts of lignocellulose, which is the primary component of plant cell walls. Refractory cellulose has become an important reason to improve the compost quality [51]. CBHA expressed in *P. pastoris* has the advantages of simple operation and low cost. The utilization of CBHA as a fermentation additive in straw composting and silage needs to be further investigated. Thus, the use of CBHA as additive for fermentation laid a foundation for improving the quality of tobacco leaves.

## Conclusion

In this study, CBHA, a Xyloglucan-specific exocellobiohydrolase, was used as a novel additive for tobacco fermentation. The contents of starch, cellulose and total nitrogen in tobacco leaf decreased significantly after CBHA fermentation. Bioinformatics analysis showed the changes of microbial community structure and composition. *Filobasidum*, *Cladosporium*, *Bullera*, *Komagataella*, etc., increased in CBHA treated group resulting the increased of relative abundance of metabolism-related functional genes, as well as the expressions of cellulase and endopeptidase. Therefore, the experiments provided an efficient, safe and environmentally friendly exogenous additives for fermentation, which had a positive effect on improving the quality of tobacco leaves.

## Supplemental Materials

Supplementary data for this paper are available on-line only at http://jmb.or.kr.
