# Supplementary material for: Effects of Exocellobiohydrolase CBHA on Fermentation of Tobacco Leaves
Source: J Microbiol Biotechnol. 2024 Jun 19;34(8):1727–37. doi: 10.4014/jmb.2404.04028 (PMC11380505; doi:10.4014/jmb.2404.04028)
Supplement: Supplementary file 1 [file jmb-34-8-1727-supple.pdf]

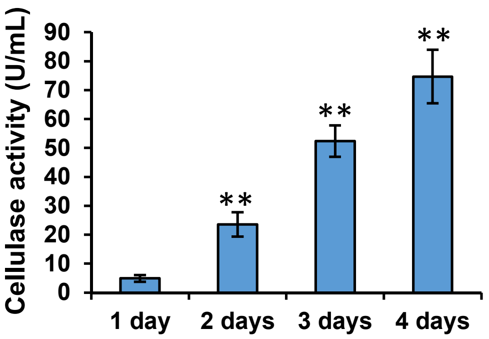

Fig. S1. The cellulase activity analysis of Pichia pastoris expression protein CBHA.

The fermentation broth was collected after induced expression. Measurement of cellulase activity of supernatant was performed through a Cellulase Activity Assay Kit according to the manufacturer's instructions. DNS colorimetry was used to determine the absorbance of the brownish red product at 540 nm by photometer. The content of reducing sugar and the activity of cellulase were calculated according to the standard curve.

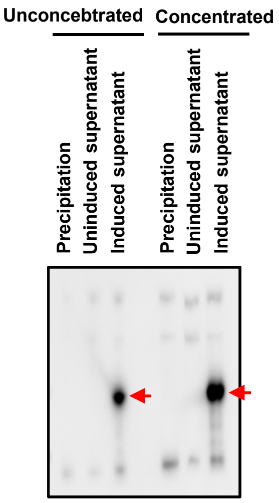

Fig. S2. Western blot detection of P. pastoris expression protein CBHA of unconcentrated and concentrated supernatant.

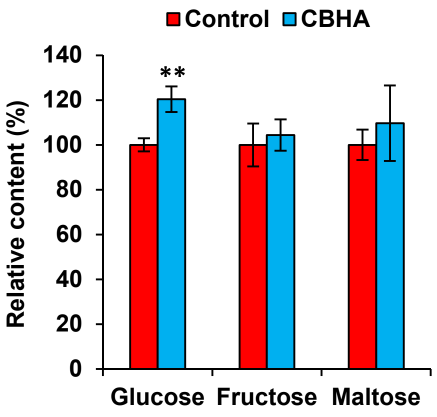

Fig. S3. Content of maltose, fructose and glucose in tobacco leaves with or without CBHA treatment.
Data are expressed as means ± SD of three independent biological replicates. Significant differences from Col-0 were determined by Student's *t*-test: *p < 0.05, **p < 0.01, t-test.

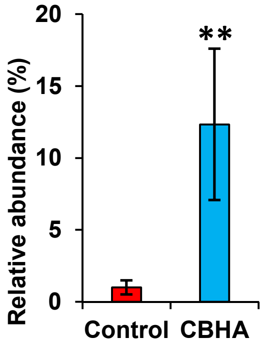

Fig. S4. The cellulase activity analysis of tobacco leaves after 48 h fermentation.

Measurement of cellulase activity was performed through a Cellulase Activity Assay Kit according to the manufacturer's instructions. Leaves were collected and frozen with liquid nitrogen and ground into fine powder. The powder was added to the reaction liquid and centrifuged to remove precipitation. DNS colorimetry was used to determine the absorbance of the brownish red product at 540 nm by photometer. Cellulase activity in tobacco leaves was calculated according to the standard curve.
